# “I Want to Be Heard”: Rape Victims’ Encounters With Swedish Police

**DOI:** 10.1177/10778012231176206

**Published:** 2023-05-29

**Authors:** Lisa Rudolfsson

**Affiliations:** Institute of Environmental Medicine, 3570 Karolinska Institute, Stockholm, Sweden

**Keywords:** interview study, justice, police, raped women, revictimization

## Abstract

Interactions with police are vitally important to victims’ ability to process their trauma. This study focused on the experiences of victims who reported a rape to police in Sweden. Thirteen women participated in interviews; the material was analyzed using inductive thematic analysis. Findings include lack of information and the role of luck in finding an understanding officer; some found comfort, and some felt violated once again. Long processing times bound participants to their trauma. Findings highlight the need for improved knowledge of trauma among police, victims’ needs for information and rights to support, and structural barriers that need to be addressed.

## Introduction

Rape is a traumatic attack on a person's freedom, dignity, and physical and moral integrity (Steenkamp et al., 2013). Previous research stress the risk of victims’ revictimization in their encounters with the police (Ahrens et al., 2010; Jordan, 2015). This introduction outlines the psychological effects of rape and victim's meeting with police in the specific context of Sweden.

### Psychological Effects of Rape

People who have been sexually assaulted are at increased risk of anxiety, eating disorders, compulsiveness, substance abuse, depression, and posttraumatic stress disorder (PTSD; Dworkin, 2020). Other studies have reported similar findings, particularly for anxiety, depression, and PTSD (Maercker et al., 2018; Pegram & Abbey, 2019). People who have been raped are six times more likely to develop PTSD than people who have not (Wemmers, 2013).

Rape has also been associated with victims’ increased fear and worry (Basile et al., 2021), self-criticism, feelings of loneliness, suicidal ideation, sexual problems, shame, and guilt (Bhuptani, 2021; Kaukinen & DeMaris, 2009). The propensity of victims to feel ashamed and guilty has been emphasized in empirical, theoretical, and clinical studies, and these feelings are often described as obstacles to victims’ reporting and seeking help (Logan et al., 2005; Wilson & Miller, 2016). Shame and guilt have also been described as central to the development of anxiety, depression, and PTSD (Bhuptani, 2021). Victim's reactions are complex, and they depend on both individual factors such as prior victimization (Nishith et al., 2000) and contextual and situational factors such as the use of physical violence and the victim's relationship with the perpetrator (Pegram & Abbey, 2019; Ullman et al., 2006). Shame and guilt are not entirely intrapsychic processes, however; they also greatly depend on the reactions and opinions of others. Myths and stereotypes about rape and rape victims often result in victim blaming and stigma (Ullman & Peter-Hagene, 2014) and can affect victims’ interactions with police (Parratt & Pina, 2017).

### Rape Victims and the Police

Rape remains one of the most underreported crimes, with only one in five victims reporting (Brooks-Hay, 2019). One reason for such low report rates may be the common perception among victims that they will be poorly treated by the justice system, starting with the police report (Shaw et al., 2017). Previous research has highlighted victims’ risk of revictimization when reporting (Ahrens et al., 2010), which may exacerbate their psychological suffering (Patterson et al., 2009; Wemmers, 2013). Many victims have expressed dissatisfaction with police responses (Jordan, 2015), and some describe interactions with police as feeling like another rape (Temkin & Krahé, 2008). Hence, interactions with police have been described as prime contributors to revictimization (Campbell, 2005; Maier, 2008, 2014).

Both patrol officers, often the first to respond, and investigative officers, who conduct the investigation, interview victims of rape (Spohn & Tellis, 2014). Patrol officers generally take the victim's initial report to decide whether to open a criminal case, while investigative officers ask victims to recall the assault in detail to determine whether the case should be pursued (Rich, 2018). Both patrol and investigative officers, therefore, have a powerful influence on the victim's well-being (Ekstrom, 2013). Because the demeanor of the police has important effects on victims’ comfort, trust in the system, and motivation to proceed with the legal process, it is important that both levels of officers show empathy (Maddox et al., 2011). Greeson and Campbell (2015) linked victims’ likelihood of continuing the legal process to police officers’ perceived level of empathy, and they found that victims who feel more comfortable in their meetings with police will disclose more information.

### The Swedish Context

In Sweden, victims of rape are assigned a plaintiff's counsel: an attorney whose purpose is to safeguard the victim's rights throughout the judicial process. This counsel has the right to participate in all police interviews (SFS §3, 1988:609). Victims may also seek support from a victim's advocate (https://www.aklagare.se/om_rattsprocessen/berord-av-brott/utsatt-for-brott/malsagandebitrade/).

The latest amendment to Swedish legislation on rape, adopted in the summer of 2018, dropped *physical resistance* in favor of *lack of consent* as the basic requirement for a rape charge. In theory, Swedish law now categorizes all sexual acts where one party has not consented as rape, defined as “forced intercourse or equally coercive sexual violation of a person who is unwilling or unable to comprehend or consent or who is in a position of dependence on the perpetrator” (Brottsbalken [Swedish Criminal Code] (1962/2018), Ch. 6, §1, author translation). Since the new legislation, convictions for rape in Sweden have increased by 75%. This increase is partly related to the new forms of sexual abuse now classified as rape, such as tonic immobility, when the victim is frozen by fear and therefore unable to resist (Möller et al., 2017). Difficulties in applying the law, however, continue to be discussed (Brå, 2020).

Both Brå, the Swedish National Council for Crime Prevention, and Swedish police officers have described sexual crimes as particularly difficult to investigate (Holmberg & Lewenhagen, 2019; Rudolfsson, 2022). Sexual crimes are often characterized by conflicting reports and a lack of physical evidence, making it difficult to meet evidentiary requirements and preventing many charges of sexual assault from being brought to court (Swedish Police Authority, 2019). Although Swedish police investigate 94% of reported rapes, only 5% of those cases lead to prosecution, and even fewer result in sentencing (Holmberg & Lewenhagen, 2019). Consequently, for many victims, the legal process starts and ends with the police interviews.

### Aim

The main focus of this study is to explore the experiences of women who reported a rape to police in Sweden, including their descriptions of their police interviews and their perceptions of the police investigations. Another focus is on the psychological consequences of those encounters—positive or negative—on the women, the women's need for support, and their thoughts on justice.

## Method

### Participants

Thirteen women aged 19–45 years participated. All participants lived in mid-sized to large cities in Sweden and all had been penetrated by one or more men during rape. Three participants had been raped just over 10 years before the interview, one 3 years prior, and nine during the year just passed. Participants’ relationships with perpetrators included partners, ex-partners, friends, acquaintances, temporary acquaintances, and strangers. All participants contacted police soon after the assault: eight called for a patrol to respond from the scene, and five contacted police within five days. At the time of the interview, five participants had their cases dropped without trial, two cases were under current investigation, and six cases had been tried in court (two convictions, three acquittals, and one ongoing with conviction under appeal). To safeguard participants’ privacy, no more detailed information is provided.

### Procedure

This study was part of a larger research project titled Female rape victims: Quality of initial police and medical care contact, funded by the Swedish Crime Victim Compensation and Support Authority (grant no: 3108/18). Other studies from the project focused on the experiences of police and medical personnel meeting with victims. The project was reviewed and approved by the regional ethical board in Gothenburg and the Swedish Ethical Review Authority (ref no: 883 18/2023-00934-02).

The author recruited participants partly through a poster shared on social media by Swedish non-profit support organizations, including Storasyster and Unizon, and partly through an advertisement and information folders distributed at gynecological emergency units in Sweden. The information described the aim of the overall project and the interview study and emphasized that participation was voluntary. It urged participants who were interested to participate to contact the author. All participants who made contact were included in the study.

All participants were interviewed face-to-face. Due to travel restrictions related to COVID-19, the first interview was conducted in the fall of 2019 and the last in the summer of 2021. Five interviews were conducted at the University of Gothenburg, and eight at conference locales in the participants’ home cities. Each interview lasted 1.5–2.5 h. The author audio-recorded and transcribed all interviews verbatim, including non-verbal information and pauses, using no qualitative analytic software.

### Question Guide

The question guide was semi-structured, allowing participants to speak freely. At the start of each interview, participants were asked to introduce themselves, state when the rape took place, and describe their relationship to the perpetrator(s). Thereafter, participants were asked broad questions about their positive and negative experiences of meeting with police, their preconceptions about police meetings with victims of rape, their needs for support, and their thoughts about justice. Follow-up questions were asked to encourage participants to give concrete examples and to reflect upon their concerns and experiences. All interviews ended with the interviewer asking participants whether anything important to them had been missed, how they felt talking about their experiences in an interview, and how they thought they would feel afterward.

The author urged participants to renew contact if they later thought of something they wanted to elaborate on or if they needed to discuss feelings evoked in the interview. Some participants chose to stay in contact by providing updates on their legal cases. Participants were also informed of their right to meet with a clinical psychologist if they needed to discuss their feelings with a professional, however, no participant used this opportunity.

### Analysis

The transcripts underwent inductive thematic analysis using a phenomenological approach to reveal participants’ experiences. This type of analysis is relatively independent of theoretical or epistemological assumptions and offers enough flexibility to allow for a range of interpretation on both semantic and latent levels (Braun & Clarke, 2006; Yardley, 2000). In this study, a bottom-up approach to identify themes kept the analysis close to the participants’ stories.

In the first step, the author read and reread each transcript in its entirety, and in the second, she scrutinized each transcript line by line and labeled all broadly relevant material with codes. The third step required rereading and sometimes renaming the codes for each interview or fusing them with others, then grouping together codes with similar content. In step four, the author and another researcher discussed the findings and combined the subthemes from each group into three preliminary main themes (pre-conceptions of police, experiences of meeting with police, and paradoxical reactions). In step five, themes were further scrutinized and compared with the original transcripts to ensure they had not been distorted, and in step six, subthemes were regrouped and the researcher agreed upon the final main themes: *Neither understanding nor being understood*, *A question of chance*, and *What about me?*

The themes cannot capture all the richness and nuance of each interview, nor are they mutually exclusive or definitive. Readers should view them as crystallized conceptualizations of the participants’ complex experiences and thoughts.

## Findings

The following text presents the main themes and subthemes that emerged in the analysis. Quotes have been translated and lightly edited to facilitate reading. Findings are summarized in Table 1.

**Table 1.** Main Themes and Subthemes.

Main theme	Subtheme
Neither understanding nor being understood	Lack of information Lack of priority
A question of chance	Finding comfort Being violated once again
What about me?	If no one else will, do I have to? When will this be over?

### Neither Understanding Nor Being Understood

In this theme, participants described receiving little or no information in their encounters with police and not understanding officers’ reactions to their trauma. This seemed to result in participants’ thinking that their case was not prioritized, and some described feeling as if they were not important or worthy of attention.

#### Lack of Information

Participants reported that police did not explain why they asked certain questions during interviews, and they were often confused by questions about how they had resisted and requests for graphic details.

“How many times would you say that he punched you?” … And, then like this, “When he put his fingers in your rectum, how long would you say he kept them there? Did he move his fingers in and out or did he hold them still in there?” and I was like, “What? Do you always ask these kinds of questions?” Well, it was very strange. I really don’t think that should have anything to do with it. I mean, I don’t think that time spent [on those details] is relevant…

Participants also talked about how hard it could be to give graphic details, as the traumatic experience blurred their conceptions: “I was like, ‘Well, I tried to focus on not dying, so my experience of time wasn’t that exact!’”

Not understanding why police asked certain questions made participants feel invaded. Although they acknowledged the abuse as rape, many participants described being uncomfortable talking about the sexual details. When police did not explain why they asked such questions, participants said they felt disbelieved, and many said that if police would explain their line of questioning it might have counteracted those feelings.

Well, I felt doubted. I mean, why won’t you [investigative police] explain why these questions are asked? And also, you need to show that, like, “I believe in you, and I know that this is really hard to talk about.” Because I felt like [any affirmation] was totally lacking.

When participants did receive information from the interviewing officer, although the questioning was still an ordeal, it was not as painful.

It's hard when they ask questions about … whether I resisted … and then, she [investigative police] was very clear with like “I have to pose this question because, well, this is the law.”

Participants also said they were not informed about how their case was being investigated. Without this knowledge, many felt their police interviews were like a game in which they had to guess what investigative measures the police might or might not have initiated. Participants also seemed unnecessarily confused when police did not inform them of their right to legal counsel. Being uninformed, especially when their case did not go to court, made participants feel that what had happened to them was not important to police.

They [police] sent me a copy-paste letter, referring to legal principles. Well, “it's she-said-he-said about events,” and, “even if new evidence should surface, that wouldn’t change this decision” or something like that.

When participants were not told why their case had been dismissed, they wondered whether they had done something wrong and wished they had understood what the police had been asking of them: “I don’t know what their [police] responsibilities are, but I mean [sighs] information about—what? What was it they wanted from me?”

#### Lack of Priority

All participants described having a preconception of Swedish police not prioritizing reports of rape against women. This made them fear that the police would not believe them and that they would have to meet police expectations during interviews. Participants described being frustrated by long waits for updates on the preliminary investigation, which sometimes made them wonder if those waits were a sign that police did not believe them or forgot about them. Many expressed anger toward the Swedish justice system for not prioritizing and allocating resources to safeguard the rights of raped women.

Because that's one of the things I get angry about, when people talk about the principle of legal security, especially regarding rape … and offenders are acquitted because “no innocent shall be found guilty.” However, what about the principle of legal security for the victim? When perpetrators are acquitted—and you know that they committed the crime. In addition, all [rape] cases that are being dropped—in what way is that legally secure? I feel like that's the opposite.

Many participants felt police had not investigated their cases thoroughly. They described how police had not contacted witnesses, obtained results from the forensic medical examination, or gathered surveillance that could have helped their case, and further, how police had lost important evidence and made detrimental errors. Participants described that when the interview was not video- or audio-recorded, the hand-written summaries often contained factual errors such as the wrong date, distorted their stories, and failed to record important information.

Well, not to include everything. I mean, the summary was like this brief, and it was … I told them lots of things that weren’t included … Yes … everything was handled so poorly [giggles with tears in her eyes] I realize that as I talk about it [cries]…

Participants who contacted police immediately after the crime talked about how hard it was to describe what had happened as they were in shock. Some described part of the trauma of rape as losing their language—becoming “dumb.” When participants did not feel safe during questioning, it was even harder for them to give detailed records. The inability of police to understand how shock and trauma affected participants’ ability to describe what happened made participants wish that police were required to have more knowledge of psychology.

I think that is a huge problem, that they [police] don’t know … they lack knowledge. They, oh there's so many things … and especially if you report very soon after the abuse … at that time, your function is really weird.

When police did not seem to understand their loss for words, participants felt disbelieved and invalidated.

I couldn’t talk about it, and I’m afraid that she [investigative police] interpreted that to mean it wasn’t that bad. Like, because I couldn’t account for what happened [her interpretation was] that I didn’t care enough about it. … This is so hard [teary eyes]. I’ve read the statement and it makes me so angry. It said something about me saying that it hurt because he bumped into my cervix and then she [police] asked “with what?” and then [the statement] says that I couldn’t answer the question. Like, seriously? [cries]

Errors in interview records made participants wish for a second interview, and some made continual attempts to be reinterviewed or to be able to amend distorted notes. When police did not allow them to ensure that their stories were recorded correctly, participants felt that police dismissed the importance of their cases and prioritized other tasks.

Why can’t they [investigative police] just … it wouldn’t take that long, right? Just allow me to come down [to the station] and say once again what happened, so it's corrected. … I don’t understand why they can’t, because I told them “There's a lot of errors in this.” Why didn’t they allow me a second interview? It wouldn’t take more than half an hour to record it…

Participants who expressed fear about what the perpetrator would do when he discovered that they had contacted police talked about how police did not prioritize their safety. Those participants described how police sent them around to different officers and different departments, without anyone being able to make them feel safe.

There's a lot of things … if he hadn’t been sentenced, I really didn’t feel protected … or supported by police. Not at all.

### A Question of Chance

In this theme, the quality of participants’ treatment varied within and between participants’ stories. No matter how their case had been handled, participants spoke about how things could have been worse, and many described police handling of their reports as a question of chance, of meeting the “right” officer and being the “right” kind of victim.

#### Finding Comfort

All participants said they felt comforted when police validated their experiences. Many had worried that their complaint would steal police time from someone more in need of attention. When police validated them, participants also reflected on police objectivity. Many thought that police were not supposed to show empathy but were grateful that they did.

They’re [police] supposed to be objective, but the patrol officers really validated my experience … I don’t know if they’re supposed to act that way as police, but it really helped me. … I don’t know if they’re allowed to, but their constant validation felt good, like, “you’ve done the right thing” and to be reminded of that. Like, “you’re doing the right thing reporting this.”

Participants described how hard it was to admit to themselves that what happened was in fact rape—preferring to talk about it as sexual abuse or sexual violation. When police used the term rape, it comforted them and helped them to grasp what had happened.

[I said] “This isn’t rape … I’ve been sexually abused,” and she [patrol officer] said “Listen, that's not it, you can’t call it that.” … I know she said that multiple times. “Stop calling it that—this is rape.”

Some participants described feeling more comfortable with a woman officer, and many speculated that police made sure to send a woman officer to a woman victim. Some also said that police had asked them if they preferred a woman to meet with them and made male patrol officers wait outside.

Four patrol officers, one woman … she came in [my apartment] first, and she said, “Do you want to … the other officers are men, and if you like they can wait outside on the stairs,” and then they [male police officers] waited there.

Although some participants described meeting with empathetic and engaged investigative officers, many described patrol officers as more tuned in and caretaking. Some speculated that being a patrol officer meeting with victims in their emotional states made those officers more empathetic and present. Some also reflected that patrol officers were often younger than investigative officers and that young people had a more current perception of the full extent of sexual violence.

I don’t think it's the level of experience either, how you’re supposed to meet victims, but rather … I think it might be that younger officers do it better. Cause … I think it [sexual violence] is more acknowledged and talked about nowadays.

Participants who described comforting encounters with police also reflected on the injustice of knowing that police did not meet other women in their situation in the same way. For some, such reflections seemed to result in kind of a survivor's guilt.

And I’m supposed to juggle that and handle being grateful that my … experience [with police] is good. That's like saying I should be happy about what happened as well—I feel some kind of guilt toward those whose experience [with police] hurt them.

#### Being Violated Once Again

Some participants regretted reporting to police, elaborating on the symbolic importance of taking a stand, by reporting, and the psychological cost of the process. Participants described situations in which police conveyed stereotypical perceptions about both victims and perpetrators of rape.

My mom called [the police station] and said, “Hi, I’m not sure where, but my daughter has been the victim of a gang rape,” or maybe she said, “She has been raped by several men,” and “Where should we turn to for help?” The police asked my mom, “Yes, those [perpetrators] were immigrants, right?” and my mom said, “No, they were Swedish men,” and he was like, “Okay.”

Participants described how police were not tuned in to the vulnerable and exposed state of a raped woman. One participant described how police questioned her level of fear since the perpetrator had not seemed “big and strong,” and many described police making offensive comments about their cases. Some described feeling cheated into thinking police would offer them more help than they did: “And you feel stupid, like you’ve been duped … they [police] are supposed to help you, but….”

Many described the interview rooms at the police station as unsuited for victims to feel safe telling their story. One participant even compared the interview room to a “morgue.” Some participants discovered during the investigation that the perpetrator had taken pictures or filmed parts of the assault. They felt humiliated when police made them watch themselves during the attack, and many described that they felt doubted and disbelieved. Some described how police also seemed stressed while watching such material, and some attributed offensive comments to the officer's own stress levels.

He [investigative police] was under the impression that … this guy [perpetrator] was into a certain type of sex that maybe everybody doesn’t like. And he [police] told me, while we were watching this [video evidence], or right before, and I remember getting angry and saying, “What do you mean? Even if he likes it rough, there's still a prerequisite for consent, right?” And then, he was like, “I didn’t mean it like that.” Just like me, he was stressed, and he tried to make conversation, kind of.

Many participants described having to tell their story repeatedly as officers were often replaced during the investigation. They talked about how the investigative officer could call them at any time, asking for more information and making them relive the details of their trauma. Staying open to the possibility that police could call them at any time made it hard for participants to let go of their trauma, even for a short while, as they were forced into a constantly activated emotional state.

It's like … it never leaves your mind. Cause when you least expect it, you get one of those letters or a call from police [whispering] it's crazy…

All participants described the difference between meeting with a woman or man officer. Many said they believed that a man would not understand, and that in any case, they were uncomfortable discussing the details of their trauma with a man. Many participants, therefore, asked to meet with a woman officer, but when that woman was not understanding, participants felt duped and even more violated.

And never have I felt so belittled and violated, because this officer was a woman, and I thought that she would have more [empathy]…

In many interviews, a story unfolded where the violations caused by non-empathetic police seemed to intertwine with the trauma of being raped. One participant described the police interview as a continuation of the “trauma creation” that began with the rape. Some perceived that the Swedish justice system was biased in favor of the perpetrator, and many described how the negative reactions of others, especially police, lingered as a non-healing wound that felt like “being raped once again.” Some even said their experiences with police caused them the most hurt.

In some ways, it's like, the way this has been handled by the judicial system, I mean, that has affected … in some ways, that has hurt me even more…

### What About Me?

In this theme, participants described how they came to feel responsible for their own police investigation, for gathering evidence and arguing for the importance of their case. Long processing times made participants feel unable to process their experiences. Participants also described how their experiences had changed them and altered their perceptions of safety and sexuality, and they stressed the importance of telling their story in the hope that it might help others.

#### If No One Else Will, Do I Have to?

Many participants described feeling responsible for their own police investigation. Some described trying to argue with police about the importance of pursuing their case. Many described gathering evidence, contacting potential witnesses, saving bed sheets, and in some cases, trying to get a confession from the perpetrator through social media or other means. For some, negative preconceptions about how police handle reports of rape seemed to make them hesitant to contact police before gathering evidence themselves.

I thought, “If I’m going to report this, I need to get it in writing”, so I hung up [phone] and wrote to him [perpetrator] on Messenger instead.

Participants described using Google to find legal aid and emotional support, and some described trying to appeal an acquittal when the prosecutor did not. For some, it seemed as if their motivation to gather evidence and pursue their own investigations overshadowed both their need to process their experience and their ability to move on. Some participants described developing a sensitivity to the least indication of others disbelieving or blaming them. For example, being questioned as to why they had invited the perpetrator to their home or followed him home made participants immediately decide that the other was against them. Some described becoming suspicious when their case was dropped or when the perpetrator was acquitted, thinking maybe police and prosecutors were biased in favor of the accused.

[Investigative police said] “Well, this is going to be dropped in two weeks” … and then that's exactly what happened … then you become like paranoid, “Are they in cahoots with … do they know the perpetrators? Is the prosecutor…?”

Many described trying to contact the investigative leader through repeated calls to receptionists or trying to find legal counsel through various organizations outside the Police Authority. Participants said such efforts were exhausting and made them feel like an inconvenience. Not only did participants talk about feeling responsible for police and legal tasks, but some also described how others had conveyed that receiving justice was the participant's own responsibility.

Like, I know that they mean well, but like this “Don’t give him [perpetrator] that your life is ruined.” I mean, “Of course, I don’t want that!” but it's really hard, it's the last thing I want—to give him [perpetrator] more of me.

Some described creating their own justice, for example, by contacting the perpetrator's wife and letting her know what happened. Many said the best revenge was for them to be happy and continue with their lives, not letting what happened affect them. However, justice in the form of moving on seemed a heavy weight to carry.

I constantly try to think … like, “The best revenge is that I’m alive” [giggles, then cries ]. … And I try to believe in that, but that's harder. I try to think, and to [sighs] keep going … and like [sniffs], I won’t [sighs] … fall back into … destructive patterns, but rather … the best revenge is … to live my life as well as I’m able to [voice cracks].

#### When Will This Be Over?

Participants’ spoke of the excessive time spent not only with police but also with the hospital's psychosocial support and staying connected with their legal aid. Many described having been forced to make their experience known at work, as they had to explain why they were absent when attending such meetings. Such disclosures sometimes seemed to force participants to put up a façade, which often hindered them from reacting naturally. For many, letting others know what had happened to them also seemed to make participants more vulnerable to others’ negative reactions. Many described how others had told them it was time to let go and move on long before they were ready to. “They [friends] stopped talking to me. They didn’t want to hang out with me if I couldn’t be happy.”

Participants described the negative effects of long processing times with police, even when they understood that the prosecutor would probably not pursue their case. Some speculated that police did not care about reports of rape but, instead, focused on less serious offenses. Participants also described how long processing times prevented them from processing the psychological consequences of what happened. They talked about their traumatic reactions as putting them in a regressive state in which they struggled to meet their most basic needs. Some expressed frustration over these lingering reactions and the hurt that did not seem to go away.

It's like, you go back to being a child—suddenly eating is work, you must gather the strength to take a shower, you’re supposed to … sleep. You’re back at square one, getting the most basic, like, what we [usually] do automatically suddenly seems to have so many steps to make it work … I get really mad at myself sometimes, like “Why can’t you just go to sleep?” or, “You need to get up in the morning, go take a shower, just eat!”

Participants also described how long processing times bound them to the traumatic event, never allowing it to slip their minds. Some seemed to place the blame for being unable to smile and let go on themselves.

There are so many times I’ve thought, “Why can’t I just repress this, forget, and move on without reporting it?” Then I wouldn’t have to be in the middle of it all the time. … I struggle with that daily. “Why can’t I just feel fine?” You put a lot of the blame on yourself.

Participants described how their experiences had changed them. Many described an increased fear of men in general, and some said their sexuality would probably never be the same again. Some talked about the importance of healing themselves through new sexual experiences, while others said it was impossible to engage in new sexual activity. They described having nightmares and being afraid of both crowded and empty places, making some avoid taking public transportation or being out late at night. All described being triggered by references to rape in TV shows and popular culture, and many described needing the perpetrator to understand the full depth of their hurt, and how it affected participants’ relationships with themselves and with others.

That is something that can … really … I mean, it lingers in my mind at night, when you can’t sleep and your life turned out this way … and that is, he [perpetrator] will never understand … He will never understand what he's done to me. He ruined my life. That doesn’t mean that I won’t take it back, but he ruined it. All my relationships were totally ruined, cause people still don’t know how to handle these things … my co-workers and my closest friends [voice cracks]. That's something he will never understand.

Only a few described justice in terms of convicting the perpetrator. Rather, participants spoke of justice as being listened to, having their experiences validated, and being allowed to react as they needed to, even when they knew it would be difficult for others to understand. When asked about their motives for participating in the study, participants described their need for spaces in which they could talk about the psychological impact of police encounters, both positive and negative, as well as the impact of others’ reactions. Participants described being able to tell their story as important for their own healing, and they wished that their experiences would help other women in their position.

Oftentimes, you feel silenced and you feel like you’re not being heard. Even though it [rape] is talked about, there are so many pieces in it that hurt—and you’re not prepared for that. … I want to be heard, if not for me, then for the sake of others.

## Discussion

This study focused on the experiences of women who reported a rape to police in Sweden. The findings both illustrate the participants’ vulnerability and psychological trauma and demonstrate their courage and resilience. The interviews with these women were colored by sadness and anger, but also by their hard work in safeguarding their rights within and apart from the judicial system.

In previous studies, victims report that being treated fairly and respectfully by police was as important to them as the legal outcome of the case (Jordan, 2015). This study found that police were arbitrarily assigned to rape victims and often did not adequately inform them of their rights or the progress of their case. While some women were assigned a sympathetic officer who might comfort them during the police meetings, others felt revictimized. This discussion focuses on how to help victims feel safe, understood, and respected in police interviews, and focuses specifically on the importance of police knowledge about trauma, victims’ needs for information and support, necessary attributes of police officers, and structural barriers to victims’ reporting and continuing to participate in the investigation.

### The Importance of Police Knowledge About Trauma

Sexual assault is known to affects victims’ short- and long-term mental health, with PTSD being the most studied long-term consequence (Maercker et al., 2018). Victims often report changes in their normal integrative and cognitive functions during and immediately after the traumatic event that impair their consciousness, memory, self-identity, and perception of the environment (American Psychiatric Association, 2013; Garcia-Esteve et al., 2021). Acute stress disorder (ASD) refers to the initial psychophysiological responses that appear 3 to 30 days after a traumatic event, resulting in significant functional impairment (American Psychiatric Association, 2013). In a recent study by Garcia-Esteve et al. (2021), two-thirds of rape victims showed signs of ASD, and up to 75% continue to show clinically significant post-traumatic, depressive, or anxiety symptoms six weeks after the assault (Short et al., 2021).

Previous studies show the importance of trauma-informed police responses (Rich, 2018) and of police training in the neurobiology of trauma (Campbell, 2006). Police require specialized training to identify and adequately respond to victims’ psychological reactions, particularly PTSD, shame, self-blame (Spohn, 2020), and ASD. This study's findings highlight how important a victims’ feeling of safety during the interview is to their ability to describe the assault. Stress impairs memory retrieval, and victims’ recall of details depends on their emotional stability, comfort, and trust in the interviewing officer (Rich, 2018). The safer a victim feels with the interviewing officer, the less stressed they will be, and the more information will likely be retrieved (Hopper et al., 2020).

In this study, participants reported that the trauma of the rape blurred their conceptions, making it difficult to give graphic details and keep track of time, especially if they were interviewed soon after the rape took place. Some also described that during their first interview, the immediate shock made them misconstrue the sequence of events. Previous research on rape interviews and the neurobiology of trauma suggests conducting a second interview as memory can improve over time and may make it easier for victims to remember details (Hopper et al., 2020). Participants in this study, however, said they were allowed only one interview and that their story had been incorrectly recorded. In Sweden, leaders of the preliminary investigation are responsible for how interviews and interrogations are documented (RPSFS, 2000:62); protocols are to be followed in a way such that they give a credible picture of the events described (SFS §22, 1947:948). In this study, participants described sometimes noting detrimental errors in the hand-written transcripts of interviews and the difficulties of having such errors corrected. Previous research has called for police to avoid compiling final statements prematurely and to allow descriptions to evolve over time. Police can address inevitable contradictions later in the process and should address those contradictions in the spirit of clarification rather than disbelief (Horvath et al., 2011). There is no scientific basis for assuming that a victim's later, more complete, memories are less credible than her earlier, less complete memory of events (Hopper et al., 2020). Consequently, it seems urgent to both address victims’ ability to correct errors in early interviews and written documentation and to review the protocol of hand-written interview reports instead of making it standard to audio/video record all interviews with victims.

The findings of this study also indicate that victims may exhibit paradoxical reactions in their encounters with police: sometimes insecure, passive, and sad; sometimes angry, intent on gathering evidence, and arguing for the importance of their case; sometimes grateful that things were not even worse. Police must understand that such inconsistent reactions to trauma and crisis are common, and they must resist resorting to stereotypical images of how a victim is “supposed” to react. In this study, one participant's inability to give graphic details resulted in the interviewing officer's distrusting her and failing to record her story correctly. Police officers who interview rape victims need to have patience and to tolerate changeable reactions. Inappropriate affect, topic switching, avoidance of eye contact, and apparent forgetfulness can all be the result of trauma (Hardy et al., 2009; Rich, 2018).

Neurobiologically, memories of emotionally significant details are enhanced during sleep, which also reduces stress (Payne et al., 2012). Previous Swedish reports suggest that victims need one night's rest before being interviewed (Swedish Justice Department, 2005), and many professionals call for allowing victims two full sleep cycles before conducting in-depth interviews (Hopper et al., 2020; Payne et al., 2012).

The findings of this study indicate the need for Swedish police to increase victims’ feelings of comfort and safety during interviews and to acquire more knowledge about trauma. Addressing these issues could both elicit better information and reduce the risk of non-empathetic and victim-blaming responses to victim's reactions.

### Victim's Need for Information and Support

A main finding in this study was the victim's lack of information. Participants described not understanding why certain questions were asked, what investigative measures were taken, and why their case was dropped without charge. A previous Swedish report (Swedish Justice Department, 2005) stressed the victim's need to be informed about the judicial process and how the police are investigating their case. Previous research has also stressed the importance of police explaining each aspect of the process, why certain questions are asked, and that it is acceptable if the victim is unable to answer all the questions (Brown et al., 2010). This study indicates that when victims understand why questions are asked, their psychological stress and risk of self-blame decreases. Participants who were given information still described the process and interviews as an ordeal, but they did not seem as hurt; they perceived the interviewing officers as more empathetic and less doubting.

Victims of rape lack control during the assault and over their subsequent physical and psychological reactions (Möller et al., 2017). Being uninformed and not understanding the judicial process can trigger emotions connected to the powerlessness participants experienced during the rape. Research on interpersonal violence and stress has found that stress reactions are easily aroused in a traumatized individual (Foa et al., 2019). Situations perceived as unpredictable increase victims’ stress, while predictability decreases it; knowing what potential discomfort lies ahead has therefore been associated with lower stress (Sapolsky, 2004). In this study, lack of information made participants feel blamed, doubted, and sometimes responsible for their case being dropped without charge, which they sometimes attributed to their own failure to understand what police wanted from them. Lack of information may therefore be a risk for victims’ blaming themselves, and further, injuring their mental health.

In Sweden, only 7%–11% of reported rapes are cleared, which is comparable to rates in other European countries (Holmberg & Lewenhagen, 2020) and in the US (Spohn & Tellis, 2014). However, in this study, rather than securing a conviction for the perpetrator(s), participants were more invested in being heard, validated, and allowed their own psychological reactions. This priority is in line with procedural justice (being treated fairly with dignity and respect and feeling heard throughout the process; Hohl et al., 2022) and the feminist concept of kaleidoscope justice (a complex, multifaceted, and fluid perception of justice within the lived experience of rape victims; McGlynn & Westmarland, 2019). Perceptions of procedural justice in police encounters have been found to influence victims’ self-worth, value, trust in the police, and willingness to cooperate with the investigation (Hohl et al., 2022). Furthermore, the presence of victims’ advocates has been reported to both decrease victims’ stress and improve their treatment by police (Campbell, 2006). In Sweden, victims of sexual crimes have the right to legal counsel to safeguard their rights throughout the process and assist through legal aid (SFS §3, 1988:609). In this study, however, many participants had not been informed of this right. Participants who had been filmed or photographed during the rape would have particularly benefitted from this support when shown such material.

The findings of this study indicate an urgent need to improve the information given to victims on how their case is being investigated and why certain questions are asked. Furthermore, victims need to be informed of their rights to legal aid and support and to have that legal counsel or a victims’ advocate present during interviews. Ensuring victims receive such information could decrease the risk of their blaming themselves and increase their sense of justice and willingness to cooperate during the investigation.

### Attributes of Police Officers and Structural Barriers

Typically, victims of rape can choose neither which patrol officer to report to nor which investigative officer will handle their case (Spohn & Tellis, 2014). As a result, reporting rape has been described as “playing the police lottery” (Jordan, 2011, p. 245). In this study, participants said that meeting with an empathetic officer and having their case investigated properly was a question of luck.

This study found that victims did not expect sexual crimes to be a high priority for either the Swedish Police Authority or the Swedish justice system, and they reported that their rights as victims were neither communicated nor met. Police officers need both training and structural prerequisites such as an appropriate place and adequate time to meet effectively with victims (Spohn & Tellis, 2014). In previous studies, Swedish police have described their own wish to be empathic and supportive, but they also described lacking the necessary training, infrastructure, and support needed to stay motivated, working in a headwind, and trying rather than succeeding (Rudolfsson, 2022). In Sweden, allegations of rape are generally investigated as serious or domestic crimes. However, previous studies in Sweden report that rape allegations compete with high numbers of gang-related shootings and are therefore often less prioritized. As a result, in some parts of Sweden, less experienced officers are assigned to investigate rape charges (Backhans et al., 2021). It is well established that the complexities of rape investigations and the needs of victims demand a specialized response (Jordan, 2011). For example, officers with higher educations are less likely to endorse rape myths that stereotype damaging and dated ideals of masculinity, supporting men's violence against women (Parratt & Pina, 2017).

This study also shows the negative effects of prolonged processing times. Shorter processing times increase the chance of resolving allegations and decrease the psychological stress of the legal process on both victim and suspect. However, in Sweden, long police processing times are reported—sometimes attributed to rape cases being less prioritized (Holmberg & Lewenhagen, 2019). Specialized departments for sexual crimes have been shown to shorten processing times (Backhans et al., 2021), indicating a need for more such departments in Sweden.

Participants described how staying available to answer police calls for additional information kept them bound to their trauma, added to their stress, and prevented them from psychologically processing what happened. Participants in this study also described how their case had been handed over to different officers. This forced victims to repeat their story, increased the risk of investigative errors, such as misplacement of evidence, and seemed to add to victims’ psychological stress.

Participants in this study described patrol officers as more tuned in, present, and empathetic than investigative officers. Empathy has been stressed as an essential resource in meeting with victims, although it can also increase officers’ risk of internalizing problems (Rudolfsson & Sinani, 2022; Tone & Tully, 2014), also known as secondary traumatization (Greinacher et al., 2019). Secondary traumatization is a risk not only to officers’ own health, but also to their ability to treat victims with respect and sensitivity (Turgoose et al., 2017). Patrol officers are often younger, less experienced, and have likely had less exposure to traumatic material than investigative officers. Some of their reported higher empathy, however, may be attributed to the fact that they usually meet the victim in her most vulnerable state and at a place, possibly her own home or a hospital, where she may feel safe. Investigative officers, however, usually meet victims in uncomfortable interview rooms, which one participant in this study compared to a morgue. Swedish investigative officers have described great frustration over the lack of amenities in these rooms, such as comfortable chairs or access to refreshments for victims during long interviews (Rudolfsson, 2022).

In many countries, rape interviews are assigned to officers of the same gender as the complainant (Spohn & Tellis, 2014). In Sweden, there is no such formal assignment, but the findings of this study indicate that women victims are more comfortable meeting with a woman officer, and previous Swedish studies have described assigning women officers to women victims as “common sense” (Rudolfsson & Sinani, 2022, p. 434). Reported rapes are overwhelmingly committed by men (Brå, 2021), and male officers may trigger trauma in women who have been raped (Temkin & Krahé, 2008). Findings from this study indicate that victims expect more empathy and understanding from women officers, but if women officers are not perceived as understanding, the psychological harm to the victim increases. Previous research has described the risk women officer run of over-identifying and becoming too personally identified with the victim (Rudolfsson & Sinani, 2022). However, there is also a risk of women officers deliberately under-identifying to distance themselves from the image of “woman as a victim” (Jordan, 2002, p. 336). Regardless of gender, experience and specialist training may improve officers’ sensitivity and compassionate treatment of victims (Jordan, 2002). The complex and challenging nature of rape investigations demands higher levels of knowledge and training for investigative officers (Holmberg & Lewenhagen, 2019; Jordan, 2011), and the informal Swedish police practice of assigning rape cases to women officers needs to be evaluated from the perspective of both the victim's wish to meet with another woman and of the importance of assigning the most skilled officers.

It seems apparent that specialized departments, with officers trained in dealing with trauma, should be created to investigate sexual crimes in Sweden, and interview rooms should be made more comfortable to allow victims a feeling of safety. Furthermore, when officers need to contact victims for additional information, simply asking if it is an appropriate time for the woman to talk could go a long way toward showing respect, building trust, and easing some of the victim's stress. These changes could shorten processing times, ensure victims are met by officers with the right training, and reduce the risk of further or revictimization.

### Methodological Reflections

Participants were recruited through advertisements at non-profit support organizations and handouts at gynecological emergency units. This recruitment method has been described as suitable for reaching those affected by the area of interest (SBU, 2016). During the interviews, the author considered it important to give participants control over what they wished to share and to allow them to present new information. The question guide was therefore semi-structured, built on broad questions related to the area of interest, and encouraged participants to elaborate freely on their thoughts.

Relationships with perpetrators and the amount of physical violence suffered varied among the participants. Previous research has shown that these characteristics may affect both the legal process (Jordan, 2015) and individual trauma reactions (Garcia-Esteve et al., 2021). Furthermore, at the time of the interview, a different length of time had passed since each participant had been raped; not only had they had longer or shorter times to process their experiences, but they had also reported to police under different Swedish laws. The decision to include all participants who volunteered was based on ethical reasons not to turn someone down. In the analysis, the aim was to identify themes pertaining to all participants, while also acknowledging the unique needs and specific context of each participant.

Thematic analysis has been described as suitable for deeper understandings of participants’ experiences (Yardley, 2000). It is a flexible method, relatively independent of epistemological and theoretical approaches. Reflexivity, however, enhances a focus on epistemology and the researcher's perception of knowledge production (Lazard & McAvoy, 2020). The first assumption of this study was that participants had important knowledge and insights into police meetings with victims of rape and how these police encounters could be improved. Their experiences are acknowledged as a valid source of knowledge and insight. However, power imbalances between victims and police, between participants and researcher, and between participants themselves, who live under different conditions and have different experiences need to be acknowledged. Furthermore, the researcher who conducted the interviews and analyzed the material was a woman, and only women participated in the interviews. This means that female lived experiences both grounded the material and provided the starting point of interpretations.

Lastly, on the importance of sharing something to eat and drink. Participants in this study described the unwelcoming environment of the interview rooms, in line with descriptions from Swedish police (Rudolfsson, 2022). During the interviews for this study, some participants became very sad and tearful. At those times, the interviewer was able to pause, offer them something to drink, and hand them a tissue to dry their tears. This small gesture of empathy and assistance contributed to the supportive atmosphere of the study interviews and seemed to make it easier for participants to tell their stories. The Swedish Police Authority should give their officers the ability to offer the same.

This study focused on meetings with police. Future studies could benefit from interviewing victims about their perceptions of meeting with plaintiffs’ counsels and the support those counsels provide.

## Concluding Remarks

In a study by Möller et al. (2017), 70% of women who had been raped reported having been unable to put up any physical resistance due to fear (i.e. freeze response/tonic immobility). In this study, while participants did describe such involuntary passiveness during the rape, they were not passive in their pursuit of justice. They stressed their need to tell their story and shared their experiences of feeling silenced. This work is shaped by the author's wish to make the participants’ voices heard.
